# A systematic review of the prevalence and determinants of nonadherence to phosphate binding medication in patients with end-stage renal disease

**DOI:** 10.1186/1471-2369-9-2

**Published:** 2008-01-31

**Authors:** Christina Karamanidou, Jane Clatworthy, John Weinman, Rob Horne

**Affiliations:** 1 Centre for Behavioural Medicine, The School of Pharmacy, University of London, Mezzanine Floor, BMA House, Tavistock Square, London WC1H 9JP, UK; 2 Health Psychology Section, Institute of Psychiatry, University of London, 5th floor Thomas Guy House, London Bridge, London SE1 9RT, UK

## Abstract

**Background:**

Cardiovascular events are the leading cause of death in end stage renal disease (ESRD). Adherence to phosphate binding medication plays a vital role in reducing serum phosphorus and associated cardiovascular risk. This poses a challenge for patients as the regimen is often complex and there may be no noticeable impact of adherence on symptoms. There is a need to establish the level of nonadherence to phosphate binding medication in renal dialysis patients and identify the factors associated with it.

**Methods:**

The online databases PsycINFO, Medline, Embase and CINAHL were searched for quantitative studies exploring predictors of nonadherence to phosphate binding medication in ESRD. Rates and predictors of nonadherence were extracted from the papers.

**Results:**

Thirty four studies met the inclusion criteria. There was wide variation in reported rates of non-adherence (22–74% patients nonadherent, mean 51%). This can be partially attributed to differences in the way adherence has been defined and measured. Demographic and clinical predictors of nonadherence were most frequently assessed but only younger age was consistently associated with nonadherence. In contrast psychosocial variables (e.g. patients' beliefs about medication, social support, personality characteristics) were less frequently assessed but were more likely to be associated with nonadherence.

**Conclusion:**

Nonadherence to phosphate binding medication appears to be prevalent in ESRD. Several potentially modifiable psychosocial factors were identified as predictors of nonadherence. There is a need for further, high-quality research to explore these factors in more detail, with the aim of informing the design of an intervention to facilitate adherence.

## Background

Cardiovascular events constitute the leading cause of death in dialysis patients, accounting for nearly half of all deaths [1]. The increased incidence of cardiac disease in patients with end-stage renal disease (ESRD) has been associated with hyperphosphatemia and more specifically an elevated Calcium × Phosphate product [2], making phosphate control an important goal of treatment. Three strategies help to control serum phosphate in ESRD: dialysis, diet restrictions and phosphate binding medication.

Adherence to phosphate binding medication may be a particular challenge for dialysis patients, due to complex treatment regimens that may have no noticeable effect on symptoms. Many patients on dialysis are nonadherent with phosphate binding medication [3] but the extent of the problem and the reasons for it are poorly understood.

There is a current emphasis in the literature on the importance of facilitating adherence to medication in chronic illnesses [4-6]. In order to develop a theory-based intervention to optimise adherence to phosphate binding medication, there is a need to first understand the factors associated with nonadherence that could be addressed. Previous reviews of predictors of adherence in ESRD have tended to focus on other aspects of the regime (e.g. diet and fluid restrictions, dialysis attendance) [7-10] and have not used comprehensive systematic search strategies [7,8,11]. This is the first paper to systematically search and review the literature specifically relating to adherence to phosphate binding medication.

The aims of this review were to assess the prevalence of nonadherence to phosphate binding medication in patients with ESRD and to identify factors associated with low adherence.

## Methods

Articles were identified for review through the search of online databases PsycINFO (1967–2006), Medline (1950–2006), Embase (1980–2006) and CINAHL (1982–2006). Papers with abstracts containing a combination of three of the terms shown in Table 1 (one from each column) were selected.

**Table 1:** Terms used in database searches ($ indicates truncation)

Haemodialysis	Adheren$	Medication$
Hemodialysis	Nonadheren$	Phosphate binder$
Uremic	Complian$	Serum phosphate
Dialysis	Noncomplian$	Serum phosphorus
Renal disease		Regimen$

Note. The search term 'dialysis' retrieved studies involving participants receiving peritoneal dialysis, therefore the papers reviewed included participants receiving both peritoneal dialysis and hemodialyis.

The database search resulted in the identification of 481 papers. Three additional papers were identified through a reference list search. Each paper was evaluated by two independent reviewers. Papers were retained if they contained quantitative studies exploring predictors of nonadherence to PB medication in ESRD, were published in English and were available from the British Library. Qualitative studies were excluded because this review aimed to quantify the number of studies reporting a statistically significant relationship versus the number of studies finding no significant relationship between each possible predictor of nonadherence and nonadherence. Papers were also excluded if they focused on paediatric adherence (patients under 18 years old), were review articles, intervention studies or case studies, or contained secondary analyses on data already included in this review.

The two reviewers extracted information on the rates of nonadherence reported and the predictors of nonadherence explored in each paper. A list of all the variables that had been investigated in relation to nonadherence was compiled. These possible predictors of nonadherence were divided into three categories: demographic, clinical and psychosocial. The number of studies reporting a significant relationship (p < .05) between each variable and nonadherence and the number of studies reporting no significant relationship (p > .05) between each variable and nonadherence were recorded.

## Results and discussion

Thirty four studies fulfilled the inclusion criteria. Key information extracted from these papers is presented in Table 2.

**Table 2:** Details of the studies included in the review

**Study**	**N Dialysis type**	**Mean age (years)**	**Mean time on dialysis (months)**	**Gender % male**	**Main statistical analysis**	**Predictors of adherence**			**Adherence assessment**	**Non-adherence definition**	**% Non-adherent**
						**Demographic**	**Clinical**	**Psychosocial**			
Bame et al, 1993 U.S. [12]	1230 HD	57	Not stated	47%	Multiple logistic regression	Age*, income*, gender, ethnicity, marital status, education	Not assessed	Not assessed	Serum phosphorus	>6 mg/dl	50%
Betts & Crotty, 1988 U.S.[42]	46 HD	Not stated	Not stated	33%	Correlations	Age, education	Time on dialysis	Response to illness	Serum phosphorus	> 5 mg/dl	74%
Blanchard et al, 1990 U.S.[38]	40 HD 40 PD	50	Not stated	48%	Correlations	Gender	Time on dialysis	Not assessed	Self report	Reporting ever missing a dose	28%
Boyer et al, 1990 U.S.[13]	60 HD	Not stated	Not stated	71%	Correlations, multiple regression	Age*, marital status*, gender, ethnicity, income, education	Time on dialysis	Social support*	Serum phosphorus	Not dichotomised	Not stated
Christensen et al, 1994 U.S.[14]	52 HD 34 PD	49	73	53%	Hierarchical regression	Age**, gender, marital status, education	Diabetic status, time on dialysis, transplant history, type of dialysis	Information vigilance, active coping	Serum phosphorus	Not dichotomised	Not stated
Christensen et al, 1995 U.S.[15]	72 HD or PD	46	73	54%	Correlations, stepwise regression	Age*, education	Time on dialysis, transplant history	Neuroticism, extraversion, openness to experience, agreeableness, conscientiousness*	Serum phosphorus	Not dichotomised	Not stated
Christensen et al, 1996a U.S.[44]	52 HD	59	62	59%	Regression	Age, education, gender	Diabetic status, time on dialysis	Neuroticism, private body consciousness, illness related physical impairment	Serum phosphorus	Not dichotomised	Not stated
Christensen et al, 1996b U.S. [16]	67 HD 14 PD	55	70	49%	Regression	Age **, education, gender	Diabetic status*, type of dialysis, time on dialysis	Perceived health competence*, health locus of control	Serum phosphorus	Not dichotomised	Not stated
Christensen et al, 1997a U.S.[33]	51 HD	57	51	59%	Correlation, hierarchical regression	Age, education, gender	Diabetic status, time on dialysis	Monitoring attentional style, trait anxiety, internal health locus of control, control appraisal, avoidant coping	Serum phosphorus	Not dichotomised	Not stated
Christensen et al, 1997b U.S.[43]	48 HD	56	65	54%	Correlations, hierarchical regression	Age, education, gender	Diabetic status, time on dialysis	Cynical hostility*, health locus of control	Serum phosphorus	Not dichotomised	Not stated
Cummings et al, 1982 U.S.[22]	116 HD	55	29	54%	Correlations, regression	Age*, gender, income, education	Time on dialysis, transplant history, regimen complexity*	Susceptibility, severity, benefits*^+^, barriers^+^, knowledge of purpose of regimen*, social support (family and friends), support from medical staff^+^, family problems^+^	Serum phosphorus Self report	>5.5 mg/dl	70% (based on serum phosphorus)
Curtin et al, 1999 U.S.[49]	135 HD	63	Not stated	47%	Chi-square	Ethnicity^###^, age, gender, employment status, education	Cause of renal failure, no. comorbidities, time on dialysis	Not assessed	Electronic monitoring (used in analysis) Pill count Self report	Overdosing/underdosing/missing more than 20% prescribed doses	73% (based on electronic monitoring)
Eitel et al, 1998 U.S.[27]	40 HD 45 PD	55	40	61%	Correlations	Not assessed	Not assessed	Efficacy expectations**, effort attributions, self control	Serum phosphorus (used in analysis) Self report Staff ratings	Not dichotomised	Not stated
Gago et al, 2000 Spain[35]	121 HD	63	37	56%	T-tests	Gender, age, living arrangements	Cause of ESRD, time on dialysis	Knowledge	Not clear	Not clear	46%
Hilbert, 1985 U.S.[29]	26 HD	47	54	35%	Correlations, ANOVA	Age, income, education, social class, religion, gender, significant other	No. times hospitalised, time on dialysis^+^	Directive guidance social support^+^, affection social support	Composite self report scale – adherence to medication, fluid and diet (used in analysis) Serum phosphorus	Not dichotomised	Not stated
Horne et al, 2001 U.K.[17]	47 HD	49	53	49%	Correlations	Age^+^, gender, education	Duration of ESRD, time on dialysis, no. prescribed medicines	Beliefs about medication (concerns ^++^, perceived need, harm, overuse)	Self report	Those who reported sometimes, often or very often deliberately missing a dose of their medication.	26%
Leggat et al, 1998 U.S.[18]	6251 HD	58	54	50%	Logistic regression	Age***, ethnicity*, smoker*, gender, education, living arrangements	Time on dialysis, diabetic status, transplant history	Not assessed	Serum phosphorus	>7.5 mg/dl	22%
Lin & Liang, 1997 China[39]	86 HD	55	42	45%	Correlations	Not assessed	Not assessed	Health locus of control^+++, ^**	3 composite measures: Lab reports (including serum phosphorus) Self report – fluid, diet and medication adherence Nurses' assessment – fluid, diet and medication adherence	>4.59 mg/dl	61% (based on serum phosphorus)
Moran et al, 1997 U.S.[45]	56 HD	57	46	64%	Regression	Age, gender, education	Time on dialysis, diabetic status, transplant history**	Social support, conscientiousness	Serum phosphorus	Not dichotomised	Not stated
Morduchowicz et al, 1993 Israel[46]	50 HD	56	66	60%	Multivariate and stepwise regression	Education ***, ethnicity*, gender, age, place of birth, religious observance, marital status, no. children, whether accompanied to session, economic status, living arrangements	Previous PD dialysis, time on dialysis	Not assessed	Serum phosphorus	Not dichotomised	Not stated
O'Brien, 1980 U.S.[28]	63 HD	Not stated	Not stated	54%	ANOVA, correlations, regression	Age, gender, marital status^++^, ethnicity, education, occupation, type of household	Time on dialysis	Significant others' expectations regarding adherence^+++^	Composite self report scale – dialysis attendance, diet, fluid and medication	Not stated	Not stated
Reiss et al, 1986 U.S.[47]	23 HD	48	8	57%	Correlations	Family income, marital status, years married, family size, education	Not assessed	Problem solving (coordination and closure), family intelligence	Serum phosphorus	Not dichotomised	Not stated
Schlebusch & Levin, 1982 South Africa[34]	25 HD or PD	38	Not stated	48%	Mann-Whitney test	Not assessed	Organicity (cortical dysfunction)^$^	Intelligence, personality^$$^	Composite staff rating – including adherence to medication and diet	Not stated	44%
Schneider, 1992 U.S.[19]	137 HD	51	26	54%	Multiple regression	Age***, gender, ethnicity, education	Time on dialysis, frequency of physician contact	Health locus of control***	Serum phosphorus	Not dichotomized	Not stated
Sherwood, 1983 U.S.[30]	55 HD	46	48	66%	Correlations	Not assessed	Not assessed	Family understanding, family organisation*, supportive family**,^+++^	Serum phosphorus Composite self-report measure – diet, fluid and medication	Not stated	Not stated
Stamatakis et al, 1997 U.S.[20]	17 HD 4 PD	53	Not stated	48%	Anova, chi-square, multiple regression	Age*, gender, ethnicity education, occupation, marital status	Type of dialysis, cause of ESRD, transplant history	Knowledge*	Serum phosphorus Self report	Not stated	Not stated
Steidl et al, 1980 U.S.[31]	22 HD 1 PD	43	22	57%	Correlations	Not assessed	Medical assessment	Family functioning^$$$^	Composite staff assessment – dialysis attendance/medication and diet adherence	Not stated	Not stated
Takaki et al, 2003 Japan[21]	484 HD	60	98	66%	Correlations, multiple regression	Age***, gender	Time on dialysis***	Not assessed	Serum phosphorus	Not dichotomised	Not stated
Tomasello et al. 2004 U.S.[3]	129 HD 59 PD	60	46	Not stated	ANOVA	Age	Time on dialysis, diabetic status, tablet burden*	Not assessed	Self report Serum phosphorus	Reporting taking less than 80% medication as prescribed >5.5 mg/dl	38% (based on self report) 51% (based on serum phosphorus)
Tracy et al, 1987 U.S.[40]	15 HD	52	0 (starting dialysis)	67%	Correlations, ANOVA	Not assessed	Not assessed	Personality*, depression*, family environment	Composite measure – serum phosphorus and interdialytic weight	Not stated	Not stated
Vives et al, 1999 Spain[41]	31 HD	63	35	74%	Mann Whitney, Wilcoxon, T-test	Age, gender	Duration of treatment	Health locus of control	Composite score based on serum phosphorus, serum potassium and interdialytic weight	>6.01 mg/dl	Not stated
Weed-Collins & Hogan, 1989 U.S.[26]	30 HD	Not stated	Not stated	43%	Correlations	Not assessed	Not assessed	Knowledge of phosphate binders, susceptibility, severity, benefits, barriers*	Serum phosphorus	>5.5 mg/dl	64%
Wenerowicz et al, 1978 U.S.[37]	19 HD	36	7	68%	Chi-square, t-test	Not assessed	Not assessed	Health locus of control *	Serum phosphorus	>4.5 mg/dl	68%
Wiebe & Christensen, 1997 U.S.[48]	70 HD	56	141	60%	Stepwise, hierarchical regression	Age, gender, education, marital status	Diabetic status, time on dialysis	Conscientiousness, susceptibility, severity, benefits, barriers	Serum phosphorus	Not dichotomised	Not stated

Note: HD = hemodialysis, PD = peritoneal dialysis, ESRD = End stage renal disease

* p < 0.05 **p < 0.01 ***p < 0.001 relationship with serum phosphorus levels, ^+ ^p < 0.05 ^++^p < 0.01 ^+++^p < 0.001 relationship with self report adherence, ^# ^p < 0.05 ^##^p < 0.01 ^###^p < 0.001 relationship with electronic monitoring, ^$^p < 0.05 ^$$ ^< 0.01 ^$$$^p < 0.001 relationship with staff ratings of adherence

### Prevalence of nonadherence

Only 13 studies reported rates of nonadherence to phosphate binding medication. Estimates of the percentage of nonadherent participants ranged from 22–74% (mean 51%). This variation can in part be attributed to differences in the way in which nonadherence was measured and defined, for example, the mean number of people classified as nonadherent when assessed through serum phosphorus levels was 58%, compared to 31% when assessed using self report measures.

These measurement issues are discussed in more detail under limitations of the studies reviewed.

### Predictors of nonadherence

#### Demographic variables

**Figure 1:**
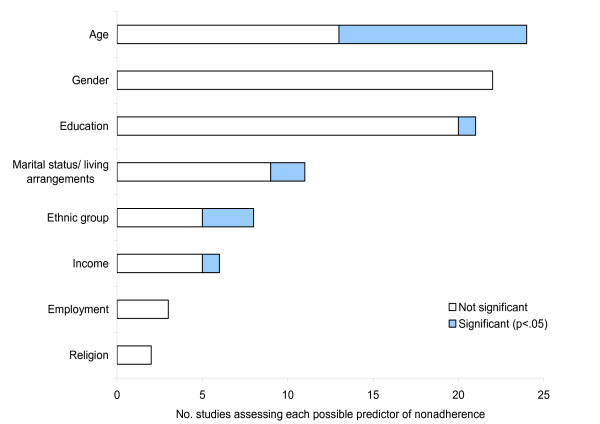
Demographic predictors of nonadherence to phosphate binding medication assessed by two or more studies.

The most frequently assessed demographic predictors of phosphate binder adherence were age (24 studies), gender (22 studies), educational level (21 studies), marital status (11 studies), ethnicity (8 studies), income (6 studies) and employment status (3 studies). As shown in Figure 1, few studies found significant relationships between demographic factors and adherence to phosphate binding medication, with the exception of those exploring the impact of age on adherence, where 11 of the 24 studies (46%) exploring this variable found a significant result [12-22]. In these studies, older age was consistently associated with higher levels of adherence. Suggested reasons for this finding are that older people may be more concerned about their mortality and have more structured lives in which to accommodate the demands of the treatment regimen [13], that younger patients may have more difficulty coming to terms with having a chronic condition [13] or simply that younger patients are more willing to report nonadherence than older patients [17].

#### Clinical variables

**Figure 2:**
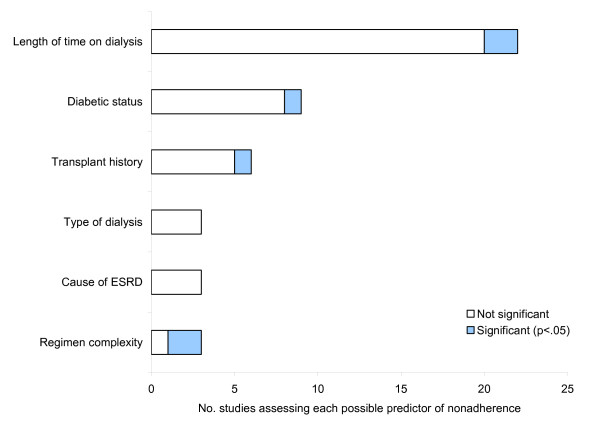
Clinical predictors of nonadherence to phosphate binding medication assessed by two or more studies.

The most frequently assessed clinical predictors of nonadherence were length of time on hemodialysis (22 studies), whether or not the patient was diabetic (9 studies) and the patient's transplant history (i.e. whether or not they had received a kidney transplant in the past; 6 studies). As shown in Figure 2, none of these clinical variables were consistently associated with adherence to phosphate binding medication. Given the large tablet burden associated with phosphate binding medication (patients on average take approximately eight phosphate binding tablets per day [3]), it is surprising that only three studies have explored the effects of regimen complexity on adherence [3,17,22], with two finding significant results. One found a correlation between complexity of the phosphate-binding medicine regimen and serum phosphorus [22] and one found that patients reporting low adherence and those above target phosphorus levels were prescribed significantly more phosphate binder pills per day [3]. Although high tablet burden may be a barrier to adherence for many patients, we cannot assume a causal relationship between high tablet burden and low adherence from these studies. One explanation is that high tablet burden leads to low adherence, but an equally plausible explanation is that low adherence results in poorer phosphate control and an increase in the number of prescribed tablets. A review across other therapeutic areas suggests that prescribed number of doses is inversely related to adherence [23] and this warrants further research in relation to phosphate binding medication. In addition, whilst qualitative and descriptive studies have indicated that the size and taste of the tablets may impact on adherence to phosphate binding medication [24,25], none of the quantitative studies reviewed explored these variables. Further research is needed to determine the role of these tablet-related factors in predicting nonadherence.

#### Psychosocial variables

**Figure 3:**
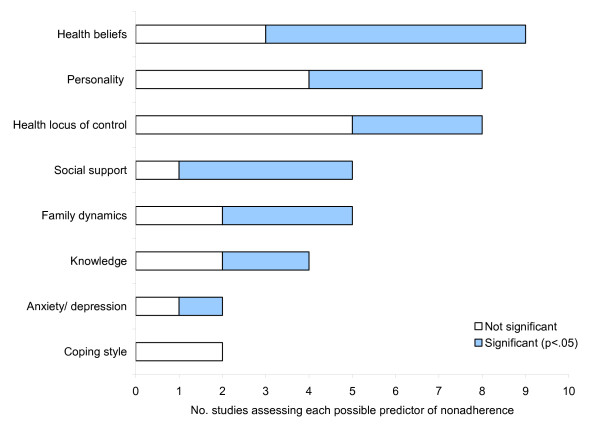
Psychosocial predictors of nonadherence to phosphate binding medication assessed by two or more studies.

Whilst psychosocial predictors of nonadherence were the least often assessed, they were more likely to be significantly associated with nonadherence to phosphate binding medication than demographic and clinical variables (see Figure 3).

Six of the nine studies investigating the relationship between health beliefs and adherence to phosphate binding medication reported significant relationships. These beliefs were all related to patients' perceptions of medication (e.g. concerns about potential adverse effects of medication [17], perceived barriers to and benefits of taking medication [22,26], perceptions of self efficacy with regard to taking the medication [16,27] and perceptions of others' expectations regarding adherence [28]). In a recent review of adherence to medication across chronic illnesses, such beliefs were identified as important potentially modifiable predictors of nonaderence that could be addressed within interventions to facilitate adherence [5].

Four of five studies found a relationship between social support and adherence to phosphate binding medication. This included support of friends and family [13,29,30] and of renal staff [22]. In addition, three of five studies exploring associations between family dynamics and adherence reported significant results. Family problems caused by the patient's illness [22], disorganisation and disagreements within the family [30] and lack of clear family structure [31] were associated with low adherence to phosphate binding medication. It is interesting that whilst marital status/living arrangements alone were not often associated with adherence, patients' perceptions of the actual support they received and the quality of their family relationships were more likely to be associated with adherence. This is consistent with findings in the broader social support literature that suggest that it is the quality rather than quantity of social support that is important in predicting mental and physical health outcomes [32].

Four of the eight studies exploring personality as a predictor of adherence to medication found significant results. Personality traits associated with nonadherence included low conscientiousness [14], high cynical hostility [33], and being expedient, venturesome, experimental and lacking self control [34].

Findings of studies looking at knowledge as a predictor of nonadherence were mixed. Two out of four studies found an association between knowledge of the purpose of the regimen and phosphate levels [20,22]. However, the other two studies found no relationship between knowledge of treatment instructions and adherence to phosphate binding medication [26,35]. Knowledge might be a prerequisite for adherence behaviour but the presence of knowledge alone may not bring about change in behaviour.

### Methodological limitations of the studies reviewed

Several methodological limitations of the studies were noted. These related to the definition and measurement of nonadherence and the study design and sampling.

#### Adherence assessment methods

A variety of methods of assessing adherence were utilised in the studies, including tablet counts, electronic monitoring, patient self-report, health care professionals' reports and serum phosphorus levels. Each method has its own limitations, as discussed in a recent review of adherence, compliance and concordance [5]. Serum phosphorus was the most frequently used indicator of phosphate binder adherence (79% studies). This can be problematic as it reflects not only adherence to phosphate binding medication but also adherence to diet restrictions and dialysis attendance. It has also been suggested that serum phosphorus levels can be affected by 'residual renal function, urine output, co-morbid illnesses, hypercatabolism, nutritional status, hormonal and acid base status, type and intensity of dialytic treatment' [36], highlighting the lack of specificity of this measure. Where studies used more than one method of measuring adherence, rates of nonadherence and predictors of nonadherence varied depending on the adherence measure used [3,22,29]. This makes it very difficult to accurately estimate the levels of nonadherence in the renal dialysis population.

#### Definitions of nonadherence

Definitions of nonadherence were inconsistent. Serum phosphorus levels that were considered acceptable ranged from 4.5 mg/dl [37] to 7.5 mg/dl [18] and this was reflected in the reported rates of nonadherence, with the study adopting the highest cut-off reporting the lowest rates of nonadherence (22%, [18]), and the study adopting the lowest cut-off reporting one of the highest rates of nonadherence (68%, [37]). Similarly, there was variation in the level of adherence that was considered acceptable in studies using self report measures of nonadherence, with definitions of nonadherence ranging from 'ever missing a dose' [38] to 'missing more than 20% of doses' [3]. More research is necessary to determine the level of adherence to phosphate binding medication required to prevent negative health outcomes.

#### Composite measures of adherence

Eight studies combined adherence to phosphate binding medication with adherence to other parts of the treatment regimen (e.g. attendance at dialysis, adherence to diet and fluid restrictions) for the analysis [28-31,34,39-41]. People may have different levels of adherence for different parts of the treatment regimen and therefore adherence to the individual components should ideally be considered in isolation. Indeed, studies that did assess adherence to different parts of the regimen separately not only reported different levels of adherence to the different aspects of treatment but also found that different factors predicted adherence to different parts of the regimen [12-15,18,21,22,26,27,33,35,42-48].

#### Study design

Only three studies utilised a prospective design [14,27,28], with the remainder using a cross sectional study design. Whilst cross-sectional studies enable the identification of associations between variables, prospective studies are required to determine causal links between potential predictor variables and adherence.

#### Sample size

Many studies had small sample sizes, with a third including less than 50 people and 6 studies (18%) reporting sample sizes of 25 or less. Only one study included a power calculation [17] and it is likely that many of the other studies would not have had the power to detect predictors of nonadherence. It is therefore possible that actual predictors of nonadherence remain undetected. Future research should ensure sample sizes are large enough for the analysis to identify significant predictors of nonadherence, should they exist.

#### Health care system bias

The vast majority of the studies were conducted in the United States of America (79%). It is possible that the health care system in the United States has unique characteristics that could influence adherence (e.g. prescription charges, private health insurance). It is therefore not possible to generalise the results to all health care systems and there is a need for further research outside of the United States.

## Conclusion

Nonadherence to phosphate binding medication is a serious problem; studies report that 22–74% patients are nonadherent with their phosphate binding medication, with the variation attributable to differences in the definition and measurement of nonadherence.

Demographic and clinical factors are not consistently associated with nonadherence to phosphate binding medication, with the exception of age (older patients are more likely to be adherent). However issues such as regimen complexity, which are likely to be important determinants of adherence, have not been fully explored and should be considered in future research.

Across studies, psychosocial factors appear to be the most promising predictors of nonadherence, including patients' beliefs about their treatment and their perceived social support. However, limitations in research design and study power create the need for further methodologically sound studies to identify the key beliefs influencing nonadherence to phosphate binders as a basis for the development of interventions to facilitate motivation, informed choice and appropriate adherence.

## Competing interests

The author(s) declare that they have no competing interests.

## Authors' contributions

CK: Literature review, first draft of paper

JC: Literature review, drafting paper, presentation of results

JW: Comments on the drafts

RH: Design of the review, structure of the paper, comments on the draft

## Pre-publication history

The pre-publication history for this paper can be accessed here:


